# Early selective attention to the articulating mouth as a potential female-specific marker of better language development in autism: a review

**DOI:** 10.3389/fpsyg.2025.1501688

**Published:** 2025-02-05

**Authors:** Itziar Lozano, Charlotte Viktorsson, Elena Capelli, Teodora Gliga, Valentina Riva, Przemysław Tomalski

**Affiliations:** ^1^Neurocognitive Development Lab, Institute of Psychology, Polish Academy of Sciences, Warsaw, Poland; ^2^Development and Neurodiversity Lab, Department of Psychology, Uppsala University, Uppsala, Sweden; ^3^Scientific Institute IRCCS E. Medea, Child Psychopathology Unit, Lecco, Italy; ^4^School of Psychology, University of East Anglia, Norwich, United Kingdom

**Keywords:** infants, selective attention, mouth-looking, autism, elevated likelihood, sex differences, audiovisual speech, language development

## Abstract

Autism is a neurodevelopmental condition with early onset, usually entailing language differences compared to neurotypical peers. Females are four times less likely than males to be diagnosed with autism, and the language features associated with this condition are less frequent in females than in males. However, the developmental mechanisms underlying these sex differences remain unclear. In neurotypical populations, sex differences in language development are also observable from early on, with females outperforming males. One mechanism underlying these sex differences may be early differences in selective attention to talking faces. During the first year, more mouth-looking generally predicts better language development, but sex differences exist. Female infants look at the mouth of a talking face more than males without penalizing looking to the eyes, and reduced mouth-looking in early infancy relates to better vocabulary in toddlerhood only in females. In this hypothesis and theory article, we propose that unique female gaze patterns to the mouth may constitute an early female-specific candidate marker that acts as a protective marker for language development also in autism. Since autism is highly heritable, investigating infants at elevated likelihood for autism offers the opportunity to search for sex-specific markers operating early in life before autistic features and language differences emerge. We argue that, as in neurotypical female infants, mouth-looking may also protect female infants-at-elevated-likelihood-for-autism population from potential later differences in language skills. If so, then sex-specific early behavioral markers, potentially acting as protective markers of language, may compensate for some genetic risk markers affecting this population. Here we gather evidence from neurotypical infants and those with elevated likelihood of autism to uncover why biological sex, the development of selective attention to the mouth, and language acquisition could be intimately related in both populations. We also propose hypotheses regarding potential sex-differentiated neurodevelopmental pathways. We end discussing future research challenges: how generalizable mouth-looking could be as a potential female-specific early language marker across contexts (experimental vs. real life), countries, and developmental time. Ultimately, we aim to target a novel protective candidate of language acquisition, informing tailored interventions that consider sex as an important source of individual variability.

## 1 Introduction

Autism is a neurodevelopmental condition characterized by difficulties with social interaction and communication, and the presence of repetitive and restrictive behaviors, as well as sensory differences (American Psychiatric Association, 2013). Approximately 1/100 children are diagnosed with autism worldwide (Zeidan et al., 2022), making it a priority research topic for child health institutions, affected children, and their families. Autism is primarily defined based on socio-communicative differences, but often entails language differences. Although not all autistic individuals exhibit language differences (Charman et al., 2003; Howlin, 2003), they are commonly observed at several stages of life (Eigsti et al., 2011; Miniscalco et al., 2012). Language differences in autism include a range of outcomes compared to neurotypical peers that often—but not necessarily—entail language difficulties at several levels, which can also be understood as adaptations. These differences can include delayed word production and comprehension (Lazenby et al., 2016), declining trajectories of receptive and expressive language (Longard et al., 2017), lower receptive than expressive vocabulary (Hudry et al., 2010), reduced production of speech-like vocalizations (Schoen et al., 2011), and reduced initiation of conversational turns (Ying Sng et al., 2018). Importantly, early language development constitutes a strong predictor of the developmental outcomes in autism (Kobayashi et al., 1992; Gillberg and Steffenburg, 1987). For example, language skills by 5 years predict long-term outcomes for autistic individuals (Billstedt et al., 2017; Eisenberg, 1956). Language is also one of the first parents’ concerns of autistic toddlers (Herlihy et al., 2015), who indeed show reduced speech vocalizations as early as 18–24 months (Plumb and Wetherby, 2013).

One of the etiological factors underlying autism is biological sex (understood here as the sex assigned at birth based on physical anatomy). A consistent finding across the literature is that autism differentially affects males and females. Females are four times less likely to present autism than males, with an average reported female-to-male ratio of 1:4 (Loomes et al., 2017). When considering the under-diagnosis of females (Duvekot et al., 2017) this ratio is closer to 1:3, suggesting that, despite diagnostic biases, females may still display autism less frequently than males (Loomes et al., 2017). Sex differences are present not only in the prevalence of autism, but also in its phenotypic features. The core features associated with autism manifest differentially in males and females (de Giambattista et al., 2021). For example, autistic females display less severe repetitive and restricted behaviors from age 4 years into adulthood and a greater awareness of the need for social interaction at ages 12–16 years, compared to autistic males (Lai et al., 2015; Park et al., 2012; Sedgewick et al., 2016; Tillmann et al., 2018). Further, the language differences frequently associated with the condition are also more frequently found in males than females between ages 1.5 and 17 years (Harrop et al., 2021; Messinger et al., 2015; but see Carter et al., 2007). Crucially, overall, these sex differences in language development are observable as early as preschool age (Mandy et al., 2012) and could potentially be present even earlier in life, as suggested by research showing delays in canonical babbling in 9-month-old males, but not females, with a later diagnosis of autism (Long et al., 2024).

However, the mechanisms underlying this sexual dimorphism in the emerging autism are not yet well understood. Multifactorial accounts that place the origins of sex differences in autism in mechanisms at various levels of analysis (i.e., sex chromosomes, hormones, brain plasticity, and early experience; Mottron et al., 2015; Muscatello et al., 2022; Werling and Geschwind, 2013) are the most common approaches, with no clear causal links between these levels. One of the most prominent and empirically supported approaches in the field is *the female protective effect* (FPE) *hypothesis* (Wigdor et al., 2022). This hypothesis posits that females require an increased cumulative load of genetic and environmental risk markers to develop autism and its associated features compared to males. Alternatively, females may carry more protective markers than males. Such protective/risk markers are usually genetically based and are thought to operate regardless of presenting or not the condition. Therefore, the protective markers affecting autistic females would also protect unaffected neurotypical females from the general population (but not autistic males or unaffected neurotypical males). Consequently, this approach opens the possibility of identifying female-specific protective markers occurring in the neurotypically developing population to shed light on those affecting autistic populations. However, the exact nature of these hypothesized protective markers remains to be fully elucidated. And, more crucially, little attention has been given to understanding which are the earliest female-specific protective markers making them less likely to develop an autistic phenotype and associated language differences. The potential candidate mechanisms operating early in life targeted so far under the *FPE hypothesis* have been scant and primarily described at a biological level [e.g., brain connectivity, Lawrence et al. (2022); neurogenetic, Jack et al. (2021)], and in relation to sex dimorphism in autism outcomes. In contrast, early behavioral markers that may be period-specific during early autism emergence, and may act as sex-specific protective/risk markers in the area of language acquisition (i.e., early markers that may be differentially expressed in females and males, or have sex-specific relations with language outcomes, or a combination of both) have been scarcely explored under this hypothesis. In particular, it is important to search for early behavioral markers for which research has previously shown both genetic and environmental contributors, allowing us to test for sex differences.

In this hypothesis and theory article, we propose that one component of social attention—selective attention to the articulating mouth—during the first year of life may constitute a female-specific early marker acting as a protective marker of language acquisition, thereby serving as a candidate mechanism underlying the FPE *hypothesis*. For this hypothesis to be supported by evidence, we would expect this mechanism to be driven by biological sex and not by likelihood of presenting autism. In the next sections, we gather evidence from both neurotypical infants and those at an elevated likelihood for autism to uncover why biological sex, the development of selective attention to the articulating mouth, and language acquisition could be intimately related in both populations. We also propose hypotheses regarding potential sex-differentiated neurodevelopmental pathways. Finally, we discuss the potential generalizability of mouth-looking as a female-specific early marker of better language outcomes across different contexts (experimental vs. real life), countries, and developmental time. Ultimately, our aim is to identify a novel protective candidate in the area of language acquisition, providing insights into potential tailored intervention needs.

Before entering into the details of our hypothesis, it is important to make some conceptual clarifications about the terms we will use throughout this paper. In the attentional literature, terms such as ‘visual attention’, ‘social attention’, and ‘selective attention’ are often used interchangeably. For clarity and to avoid misunderstandings, we will adhere to the following definitions, which, although subject to some debate, are generally accepted in the field. Starting with the broadest construct, *visual attention* refers to a multi-component complex construct encompassing alertness, spatial orienting, attention to object features, and endogenous attention (Colombo, 2001). This type of attention can be directed toward both social (i.e., involving people or social interactions) and non-social (e.g., arrows) sources of information (Salley and Colombo, 2016). The term *social attention*—which we will define further in the next section—refers to a constellation of skills that is frequently used to describe how people allocate their gaze to social events (i.e., involving people or social interactions) relative to non-social events, although this comparison has not been systematically explored in the literature (Salley and Colombo, 2016; Falck-Ytter et al., 2023). Thus, social attention would be a subtype of visual attention. Finally, *selective attention* to talking faces, which is the focus of our hypothesis, can be considered a specific subset of social attention (but note that both can be the result of a ‘selective’ process). It refers to the tendency to focus on different features of talking faces at the expense of others (usually, the eyes relative to the mouth; e.g., Lewkowicz and Hansen-Tift, 2012; Lozano et al., 2022; Morin-Lessard et al., 2019; Sekiyama et al., 2021; Tsang et al., 2018). Although this term has also been referred to as ‘preferential attention to the eyes and mouth’ (or, simply, ‘attention to the eyes and mouth’), it has been widely used in studies of visual attention to audiovisual speech in infancy. Importantly, throughout this paper we will use selective attention to the eyes and mouth, or to simplify, mouth and eyes-looking, to refer to the same construct.

## 2 Social attention: Its role in language development in neurotypical infants and autistic populations

In neurotypical development, what events infants prefer to pay attention to in their social environments seems an essential mechanism shaping what they learn. The so-called ‘social attention’ encompasses a broad, complex constellation of skills that infants use to successfully take part in their face-to-face interactions, serving as a fundamental building block for language and socio-communicative developmental trajectories and outcomes. Social attention includes, among other abilities, face processing, joint attention, gaze following, and preference for specific facial features in speaking faces—i.e., the eyes and mouth—(for a critical review of this controversial term, see Falck-Ytter et al., 2023). Crucially, this latter mechanism may have important cascading effects on later language developmental trajectories and outcomes, such that infants’ efficiency in visually attending talking faces could potentially drive better language outcomes (Bradshaw et al., 2022; D’Souza et al., 2017). What information infants prefer to pay attention to in talking faces constrains how much they benefit from processing audiovisual speech (see Belteki et al., 2022b for a review). In fact, available eye-tracking studies with infants suggest that an increase in mouth-looking takes place during the second half of the first year of life (Lewkowicz and Hansen-Tift, 2012), with infants’ mouth-looking (Tenenbaum et al., 2015) and infants’ gains in attention to the mouth during this period resulting in higher expressive language scores at the end of the first year and in toddlerhood (Lozano et al., 2022; Tsang et al., 2018). It is worth noting that the second half of the first year of life is also often when productive language skills begin to emerge, including canonical babbling (4–10 months; Oller, 1978) and word approximation (10–11 months; Laing and Bergelson, 2020). Presumably, focusing on the mouth might facilitate infants accessing temporally synchronized auditory and visual speech cues, which seems to enhance audiovisual speech processing (Tomalski et al., 2012).

Individual differences in social attention are in part genetically driven from very early on in life, as supported by recent twin studies (e.g., Portugal et al., 2024; Viktorsson et al., 2023), with selective attention to the eyes and mouth regions of faces among the most highly heritable traits (Constantino et al., 2017; Viktorsson et al., 2023). The heritability of social attention is one of the arguments why differences in this broad set of skills have been proposed as key candidate intermediate phenotypes to account for the early development of autism and the linked emerging language features before they are observable (Falck-Ytter et al., 2023; Gui et al., 2020; Jones et al., 2017). A further argument is that, in neurotypical development, genes and the environment influence social attention, and, therefore, both infants’ genetic liability for social attention and their early social experiences shape later social skills such as language (Falck-Ytter et al., 2023; Nishizato et al., 2017; Portugal et al., 2024; Viktorsson et al., 2023).

Indeed, differences in social attention—including patterns of attention to the eyes and mouth—are indicative of the core diagnostic criteria of differences in social communication in autistic individuals, though note that differences in specific social behaviors are highly variable and not systematically replicated for all (see Falck-Ytter et al., 2023 and Falck-Ytter, 2024 for a comprehensive discussion of lack of differences in orienting to faces or gaze cueing as specific illustrative examples). Given the high heritability of autism (80%; Bai et al., 2019), some of these differences in social attention also extend to infants at elevated likelihood for autism by virtue of having an older sibling with this diagnosis—henceforth, EL-infants—, compared to those without—LL-infants—(Lozano et al., 2024). Also, due to the high heritability of the condition, language differences linked to autism usually extend to EL-infants (Hudry et al., 2014). Current models of autism propose that multiple risk markers that interact during development contribute to autism emergence, as well as to the different developmental trajectories followed by EL-infants as a group, including language differences (Elsabbagh, 2020; Elsabbagh and Johnson, 2010; Gliga et al., 2014). Crucially, early susceptibilities in the developmental mechanisms underlying language development may also be present in this at-elevated-likelihood population before the different outcomes are observable, with social attention being a potential candidate.

Social attention appears to be also driven by biological sex. Sex differences in social attention—specifically in visual attention to naturalistic faces and preference for faces (versus non-social objects)—are shared by young autistic children (Harrop et al., 2018), EL-infants (Chawarska et al., 2016; Kleberg et al., 2019), and neurotypically developing infants (Alexander and Wilcox, 2012), suggesting they follow a continuum not only in autistic individuals, but also in the general population. In neurotypical infants, sex differences in social attention extend to the patterns of preference to eyes and the mouth of talking faces from early on in life. These early preferences might account for later sex differences in the developmental trajectories of early language development.

### 2.1 Mouth-looking as a potential early marker reflecting sex differences in language development in neurotypical and autistic populations

#### 2.1.1 Neurotypical population

In typically developing populations, sex differences in language development are well-documented, with females outperforming males in vocabulary size, sentence complexity and expressive language from early toddlerhood to late adulthood (e.g., Etchell et al., 2018; Marjanovič-Umek et al., 2017). Sex differences in vocabulary occur as early as 2 years of age (Lutchmaya et al., 2001; Wallentin and Trecca, 2023). One of the potential mechanisms that might underlie these early sex differences in language acquisition is infants’ visual attention to speaking faces (Kleberg et al., 2019). From birth, infants learn to pay attention to the inner features of the faces (i.e., the eyes and the mouth). Although evidence so far remains correlational, increased mouth-looking in the second half of the first year seems to potentially support better concurrent and later language outcomes by means of facilitating infants’ access to audiovisual speech cues (Belteki et al., 2022b; Tsang et al., 2018).

Crucially, Lozano et al. (2022) further explained this effect by showing that mouth-looking in talking faces might be an especially important mechanism for female infants’ language development. In a longitudinal study assessing ~100 babies, Lozano et al. (2022) showed that female infants from the general population (i.e., at the general population level of likelihood of autism) look more than males to the articulating mouth without penalizing their selective attention to the eyes, a sex-specific pattern that remained stable during the first year of life. Further, in female infants, reduced mouth-looking at 5.5 months and 11 months of age predicted better vocabulary outcomes in toddlerhood (at 24 months of age). In addition, those females with smaller increases in mouth-looking had better expressive language by the end of the first year. Importantly, these developmental relations were not replicated in males. And while increased looking at the eyes was associated with higher language scores in infants of both sexes, less looking at the mouth was associated with better language skills only in females.

This female-specific relation found by Lozano et al. (2022) contrasts with studies showing a positive relation between mouth-looking and language when combining sexes (e.g., Morin-Lessard et al., 2019; Tsang et al., 2018). The negative association between mouth-looking and language development in females, although counterintuitive, might reflect females being ahead in their trajectory of mouth-looking from early on. We speculate that it could also potentially suggest an early native-language expertise in females, as infants with better language might need to rely less on the mouth to seek visual articulatory speech cues (Lewkowicz and Hansen-Tift, 2012), and perhaps an earlier peak in mouth-looking in females, before 5.5 months. Since females looked *more* at the mouth than males by 5.5 months, they may have already benefited from this (or may be using mouth-looking information more effectively), leaving less room for later improvement, which is consistent with their better language than males at the end of infancy and in toddlerhood also observed in Lozano et al. (2022). Importantly, the timing of the negative relation found in females—less mouth-looking at 5.5 months and better language outcomes at 24 months—may be critical for interpreting this counterintuitive result. It highlights the need for investigating a wider age range, as the direction of preference and its link to language development may change across the first year of life and beyond.

Thus, although further replication is needed and this evidence was correlational (thus, should be considered as non-causal, as other non-measured factors could have mediated it), the study by Lozano et al. (2022) may suggest that sex differences specifically in mouth-looking in infancy could be a candidate mechanism driving the early female advantage in language development. More importantly, these results may suggest that, in neurotypically developing populations, selective attention to the articulating mouth in infancy may be a female-specific early marker acting as a potential protective marker in language acquisition—and, reversely, may not confer protection in language development in males, as indicated by males showing no longitudinal relations between mouth-looking in infancy and language outcomes in toddlerhood, and lower expressive language than females at age 2 years.

#### 2.1.2 Autistic population

Despite mixed findings (see Falck-Ytter et al., 2013 for a review), there is evidence indicating that autistic individuals show reduced selective attention to the mouth in talking faces as early as 22 months of age (Shic et al., 2020) and across the lifespan (Grossman et al., 2015; Righi et al., 2018; Shic et al., 2020; see Chita-Tegmark, 2016 for a meta-analysis). Moreover, although evidence is also mixed, some studies suggest that reduced selective attention to the mouth of talking faces could potentially serve as an early marker of language differences in autistic individuals [but see Åsberg Johnels et al. (2014) and Righi et al. (2018) for exceptions]. A recent study of autistic toddlers aged 10–25 months found that mouth-looking was positively associated with expressive language only in neurotypical and autistic toddlers who had acquired first words, but unrelated to expressive language in autistic toddlers who had not yet acquired first words (Habayeb et al., 2021). This highlights the complexity of the functional relation between mouth-looking and language development in autism and the need for caution before considering reduced selective attention to the mouth as a robust early marker of language differences in autistic individuals.

At least two studies have observed sex differences in social attention in school-aged autistic children aged 6 to 10 years (Harrop et al., 2018, 2020). Overall, it was found that autistic females attended more to faces than autistic males (but note that we used either silent clips or static pictures as stimuli, which may not generalize to gaze patterns to dynamic talking faces). However, to the best of our knowledge, few studies to date have investigated sex differences in *selective attention to the mouth* in autistic individuals (e.g., Ross et al., 2015). Another important unexplored question is whether, as it occurs in the general population, early sex differences in selective attention to the mouth might also account for later sex differences in language development in autistic populations [which have been found for both expressive and receptive language as early as 18 months of age, with neurotypical, autistic, and at elevated likelihood for autism females outperforming neurotypical, autistic, and at elevated likelihood for autism males; Messinger et al. (2015)].

#### 2.1.3 Current evidence in infant siblings

Partly due to the high heritability of the condition (Folstein and Rutter, 1977; Losh et al., 2008; Piven et al., 1997; Whitehouse et al., 2010), the language differences linked to autism usually extend to first-degree relatives of autistic individuals, such as EL-infants (Hudry et al., 2014). EL-infants present an increased genetic likelihood for developing the condition themselves relative to the neurotypically developing population (recurrence risk ~20%, as compared to ~1% in the general population; Messinger et al., 2015; Ozonoff et al., 2024), as well as for showing early differences in language development (Hudry et al., 2014; see Belteki et al., 2022a for a review). For example, EL-infants show lower production of consonants and fewer speech-like vocalizations (Paul et al., 2011), later onset of babbling in infancy, and delayed language production and comprehension in toddlerhood (Iverson and Wozniak, 2007). Conducting longitudinal studies with EL-infants offers a unique opportunity to advance the search for sex-specific protective/risk markers operating early in life in autism, before the emergence of the autistic phenotype and the associated language differences are observable. Thus, EL-infants allow us to investigate female-specific markers that may act as protective markers underlying *the female protective effect (FPE) hypothesis* operating early in life.

Having found that selective attention to the articulating mouth seems to be a sex-specific early marker of better language development in neurotypically developing female infants opens the promising possibility that this same mechanism also protects infant females at-elevated-likelihood-for-autism (Lozano et al., 2022). Extending the *female protective effect (FPE) hypothesis* to EL-infants, under similar genetic liability in females and males, females should be more tolerant than males to the genetic risk markers for autism, as well as to the early differences in language development. Here, we propose that it would be important to pick up the line of work by Lozano et al. (2022) on sex differences in mouth-looking in neurotypically developing infants and extend it to study EL-infants to target a female-protective candidate mechanism underlying the female protective effect (*FPE*) approach. Investigating whether these sex differences extend to at-elevated-likelihood-for-autism populations who are affected by language differences may shed light on whether sex might override or enhance genetic risk markers linked to autism. We also expect that exploring sex differences in mouth-looking as a potential sex-specific early marker of better language outcomes in female EL-infants will allow clinicians to design interventions on a very targeted skill and implement them in critical periods of early development. Ultimately, our proposal will help to mitigate language differences in EL-infants in early infancy, two years before the age of a potential diagnosis.

To our knowledge, so far, no one has studied sex differences in mouth-looking in audiovisual speech in infants at elevated likelihood for autism. Only a single study explored visual attention to static emotional pictures of faces, showing that female and male EL-infants attend differently to the mouth (Kleberg et al., 2019). However, infant siblings were tested at only one time-point, with pictures—thus, in a task that does not allow measuring selective attention to a *speaking* mouth—, and in a sample size moderate for testing sex differences (*n* = 99, 70 EL-infants and 29 LL-infants). Despite providing valuable findings, the approach taken so far has some limitations that need to be overcome by future research.

First, testing at only one time-point instead of across larger-scale time-points limits the possibility of exploring how stable sex differences are in selective attention to the articulating mouth in the first year of life. Although Lozano et al. (2022) observed stability in sex differences across infancy, charting this question in EL-infants is essential because the timing of the expression of sex differences could vary in this differently developing population. To overcome this limitation, we propose it is necessary to longitudinally track potential sex differences in mouth-looking in EL-infants and LL-infants across several time points of the first year of life and beyond. The second half of the first year is of particular interest, as it is a time period when changes in selective attention to the articulating mouth frequently occur in neurotypical infants (Belteki et al., 2022b; Lewkowicz and Hansen-Tift, 2012) and when differences in basic mechanisms emerge in EL-infants (Elsabbagh and Johnson, 2010). This approach will bring a more comprehensive developmental picture of the stability of potential sex differences in this mechanism across time, which has been uncharted so far.

Second, traditional eye-tracking tasks showing pictures as stimuli (or clips of audiovisual talking faces) have been the most common approach to testing sex differences. This way of assessment limits the generalizability of the results to natural communicative contexts. To date, sex differences in mouth-looking have not been evaluated in an ecologically valid context in EL-infants, nor neurotypically developing infants. We suggest the need to take a broader approach by tracking sex differences in mouth-looking during infancy across eye-tracking tasks. This entails testing the same hypotheses in eye-gaze patterns of infants during “live” interactions with people and when watching classical eye-tracking screen-based tasks. We believe that this cross-task approach is crucial to improving the ecological validity and applicability of the scientific findings in this area since it will inform us on how extensible sex differences are to real communicative face-to-face situations.

Third, sex differences in mouth-looking in EL-infants have not yet been assessed in a high-powered study that warrants the robustness of the findings. Studies with such a large sample size have not yet been conducted. Large-scale studies are needed to ensure the reliability of the findings, which is particularly important when investigating highly heterogeneous differently developing populations like EL-infants.

Fourth, unlike prior studies, we think it is essential to take a cross-cultural and cross-labs approach to increase the generalizability of the potential sex differences in EL-infants. If biological sex drives the trajectories of mouth-looking in infancy, and being a female protects later language outcomes in both EL-infants and LL-infants across different countries, this would support the robustness of the phenomenon of increased mouth-looking as a female-specific early marker of language operating as a protective marker.

To sum up, to overcome the limitations of previous research in the field, we propose it is necessary to longitudinally track, in a highly-powered sample of infant siblings at elevated likelihood for autism, sex differences in the trajectory of mouth-looking across the first year of life and their predictive role on later language outcomes across: (a) time-points, (b) eye-tracking tasks, and (c) countries.

## 3 Potential sex-differentiated neurodevelopmental pathways of mouth-looking and language development: Biological and environmental mechanisms

We have hypothesized as our main proposal that increased selective attention to the articulating mouth during infancy may be an early female-specific marker of language acquisition across at-elevated-likelihood-for-autism and neurotypical populations, along with a way to test it. Our understanding is that increased mouth-looking might be a female-protective attentional mechanism in itself, placed at a behavioral level. If this proves to be the case, a crucial theoretical question would be to understand the specific mechanisms through which infant females may look more at the articulating mouth than males and how reduced mouth-looking might benefit their later language trajectories. One way to address this question is to gather evidence beyond the behavioral level that points to potential early mechanisms that may be driven or modulated by biological sex in both at-elevated likelihood-for-autism and neurotypical populations, and which may play a role in later language development.

**Figure 1:**
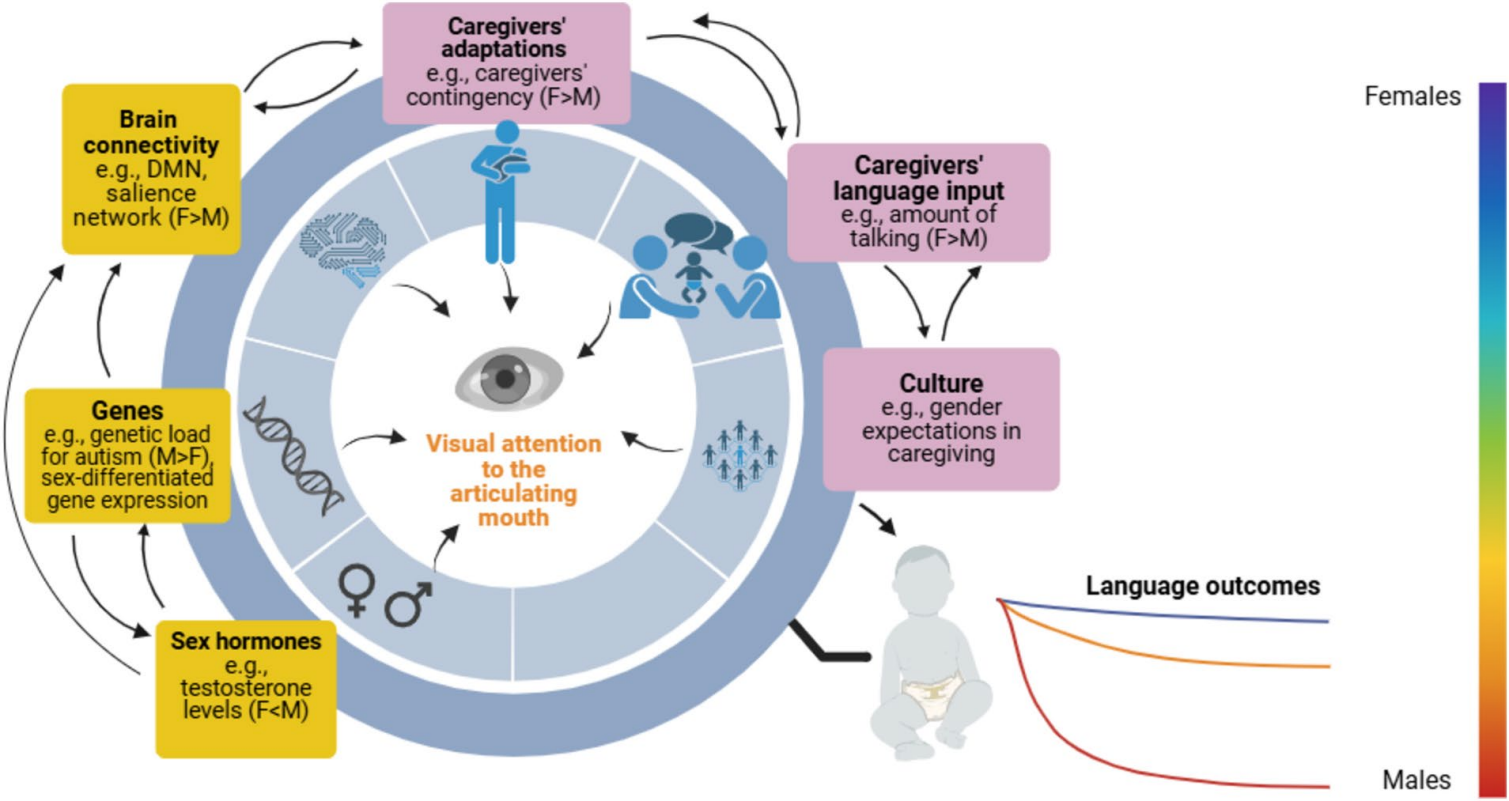
Scheme of potential mechanisms at the biological and environmental level influencing sex-specific neurodevelopmental pathways of mouth-looking and language development. Influences can be bidirectional and interplay throughout the lifespan, possibly affecting both neurotypical and at-elevated-likelihood-for-autism infants as a continuum. Female-specific protective markers may impact both infant females and males’ language outcomes at an individual level (i.e., female-predominant), but mostly benefit females at a group level. A range of potential language outcomes by sex are represented on a spectrum of colours. In the boxes, yellow represents mechanisms at the biological level, and purple represents environmental ones. F, Females; M, Males. This figure was created with BioRender.com.

Language development relies on multiple mechanisms at different levels, so it seems reasonable to expect that sex differences in the outcomes are accounted for by several mechanisms potentially modulated by sex. Once identified, we could theorize possible links between these mechanisms and selective attention to the articulating mouth (see Figure 1). In this section, we first overview early sex differences in mechanisms at a biological level that may give female infants a (primarily indirect) advantage in mouth-looking and in the relations between mouth-looking and later language acquisition. Next, we review potential mechanisms at an environmental level that could give female infants more opportunities to develop more efficient visual attention patterns to the articulating mouth and better later language skills. Because we believe that the biological-environmental distinction is a false dichotomy, as both types of mechanisms are likely not independent but interplay, we will also describe potential interactions among them.

### 3.1 Biological level

#### 3.1.1 Brain functional connectivity

Biological sex impacts early language development at several levels of analyses since prenatal stages (see Etchell et al., 2018, for a review). At the level of brain function, there is evidence of early sex differences in resting state functional connectivity already in neonates and infants, and its impact on language expressive outcomes at 1 and 2 years (Fenske et al., 2023). In a fMRI longitudinal study by Fenske et al. (2023), females showed greater connectivity between temporal and frontal areas than males. Notably, female and male neonates with greater connectivity between the right inferior temporal gyrus and the frontoparietal network—involved in establishing language and executive function processes—had better expressive language scores at 1- and 2 years. This suggests that greater functional connectivity was not female-specific, but males who showed functional connectivity patterns more similar to those of females had better later language development. Interestingly, prior studies in neurotypical infants indicate that the fronto-temporal network supports audiovisual speech integration and is functionally active already in infancy (Altvater-Mackensen and Grossmann, 2016; Dopierała et al., 2023). Moreover, although research in this area is still emerging in infancy, the right inferior temporal gyrus appears to be involved in a default mode network (DMN), which activates during tasks involving social processes, including monitoring and predicting the behaviors of others (Yeshurun et al., 2021). Because continuous audiovisual speech is a social, highly dynamic, and temporally complex stimulus (Chandrasekaran et al., 2009), we hypothesize that female infants may use the DMN network more efficiently to predict the articulatory movements of the mouth during this event.

Newborn EL-infants show different functional connectivity in the DMN network (Ciarrusta et al., 2020); however, to our knowledge, there is no evidence of potential sex differences in this network in this population. Future infant studies with neurotypical and at-elevated-likelihood-for-autism infant siblings could investigate (1) whether female infants’ greater functional connectivity in the fronto-temporal network replicates when processing audiovisual speech, (2) whether, at the behavioral level, this neural response is intertwined with greater selective attention to the articulating mouth, and (3) whether both mechanisms support infants’ ability to predict the linguistic behavior of others predominantly in females. This potentially female-specific cascading path in infancy may facilitate audiovisual speech processing, resulting in better language outcomes later in life. Sex differences in functional connectivity in other networks involved in language acquisition that are affected in infant siblings (e.g., the salience attentional network, which underlies the integration of sensory and motor representations and plays a role in orienting to salient stimuli; Liu et al., 2020) have also been found in neurotypical fetuses in utero (Cook et al., 2023), with females showing a greater magnitude of cross-region connections in somatomotor and frontal areas compared to males. Future studies should explore whether these sex differences replicate in infant siblings and whether they underlie sex-specific developmental trajectories of later language development.

#### 3.1.2 Sex hormones

At the hormonal level, pre- and early postnatal exposure to sex steroid hormones, particularly testosterone, seems to drive early sex differences in the language organization of the infant and child brain (e.g., Friederici et al., 2008; Lust et al., 2010) and later language outcomes (Kung et al., 2016; Lutchmaya et al., 2001). This is especially observable during the postnatal period of rapid changes in infants’ brain development and elevated sex hormone concentrations, known as ‘mini-puberty’, lasting between ~1st-5th month (Hines et al., 2016). For example, in an ERP study with 4-week-old neurotypical infants, Friederici et al. (2008) found that females (who are generally low in testosterone) exhibited a mismatch response (MMR) in phoneme discrimination. However, only males with low testosterone levels showed this effect. These findings suggest that as early as 4 postnatal weeks, testosterone level rather than biological sex appears to influence language function in the brain. In a follow-up study, this relation was replicated at 5 months, and, across both sexes, low testosterone and high oestradiol concentration at this early age were associated with better sentence comprehension at 4 years (Schaadt et al., 2015).

Regardless of biological sex, postnatal testosterone level also appears to influence the development of key vocal precursors of language acquisition (Quast et al., 2016; Wermke et al., 2018). For example, Quast et al. (2016) found that testosterone concentrations at 4 weeks of age negatively correlated with individual articulatory skills in babbling at 5 months. To our knowledge, no studies have tested the impact of postnatal testosterone concentration during ‘mini-puberty’ on later language acquisition in EL-infants. Only its effects on autistic traits at 18 and 24 months have been recently measured, finding no significant associations in this population or neurotypical infants (Tsompanidis et al., 2023).

Interestingly, higher prenatal testosterone is associated with a later reduction of functional connectivity between the brain’s DMN in adolescent males, but this effect is not present in females (Lombardo et al., 2020). These findings, although correlational, raise the possibility that prenatal testosterone may shape sex-specific pathways of language development through its influence on neural circuits specialized in social cognition, such as the DMN. Higher prenatal exposure to testosterone may heighten particularly infant males’ susceptibility to developing reduced functional connectivity in the DMN network, which is crucial for efficiently processing social information, including audiovisual speech, potentially affecting their later language development. Alternatively, testosterone may also shape early infants’ sex differences in some social mechanisms placed at an ‘intermediate’ behavioral level between the DMN network and later language outcomes. For example, infants’ eye contact with caregivers seems more frequent in female 1-year-olds than their male peers, and it negatively relates to earlier foetal testosterone concentrations regardless of biological sex (Lutchmaya et al., 2002). Future studies should explore these possibilities and, if so, whether they replicate across both neurotypical and at-elevated-likelihood-for autism infant populations.

#### 3.1.3 Genetic, sex hormonal, and brain functioning interactions

Genes interacting with the two described levels of analyses may also play a role in the expression of early sex differences in language development. Sex hormones, gene expression, and brain function likely interplay in their impact on potential sex-specific paths of language development across neurotypical infants and those at elevated likelihood for autism. For example, in childhood, the effect of the cumulative genetic likelihood for autism on functional brain connectivity of the Salience Network—involved in orienting attention to salient stimuli, such as faces and other relevant social events, and coordinating a response between the internal and external environment, which are key for social attention (Uddin, 2015; Menon and Uddin, 2010)—is significantly moderated by biological sex across neurotypical and autistic populations. Specifically, genetic load for autism affected functional connectivity in this network in autistic and neurotypical males, but not in females (Lawrence et al., 2022). Consistently, in neurotypical adolescents, for neural networks in which sexual dimorphism is observed (e.g., DMN, Salience Network; Teeuw et al., 2019), heritability explains up to 53% of the variation in the functional connectivity, which is partially replicated in infants (Gao et al., 2014).

Another illustration of these potential interactions is that higher perinatal testosterone concentrations increased the likelihood of language delay in neurotypical males during the first three years of life but reduced this likelihood in females (Whitehouse et al., 2012). This opens the possibility that mere prenatal exposure to testosterone concentrations does not cascade into sex-differential pathways of functional connectivity in neural networks relevant for social cognition. Instead, a distinct response to this exposure between female and male infants via its influence on cerebral lateralization may be more important (e.g., Lust et al., 2010), with high perinatal testosterone levels potentially acting as a risk marker for language development in males, but as a protective marker in females.

To our knowledge, potential interactions between genetic, sex hormonal, and brain functioning have not yet been investigated in infant siblings (but see Gui et al., 2020 for a seminal proof-of-concept on the prospective effects of DNA methylation in developmental trajectories in social attention in male infants of this population). Future epigenetic studies with infant siblings and neurotypical infants could explore whether sex modulates the relation between genetic likelihood for autism and functional connectivity in the Salience and DMN networks. Further, it would be of interest to investigate whether, in both populations, the relations between sex, genetic liability for autism, and neurocognitive networks have links ‘down’ to sex-specific patterns of selective attention to the mouth of talking faces at the behavioral level. Ultimately, it is essential to understand if these interactions modulate or influence sex-specific effects in later language skills. A recent promising finding in this direction is that early Salience Network connectivity at 6 weeks predicts individual trajectories of increased social attention (specifically, to talking faces) across the first postnatal year, and higher rates of joint attention at age 1 year in neurotypical infants, but not in EL-infants (Tsang et al., 2024). A further necessary step would be to investigate whether early Salience Network connectivity also predicts infants’ trajectories of selective attention to the articulating mouth and precursors of social communication in the first year, and whether biological sex mediates these relations and associations with later language outcomes across both groups.

### 3.2 Environmental level

Several mechanisms at the environmental level may also account for early sex differences in selective attention to talking faces and their sex-specific associations with later language development. By environmental, we mean social and cultural practices that may affect infants’ early experiences, which go from caregivers’ interactive styles to the specific properties of the infants’ languages (e.g., phonemic or rhythmic features).

#### 3.2.1 Caregivers’ language input

A primary candidate is sex/gender^1^-dependent parental style during interactions. Caregivers’ language input may differ based on infants’ biological sex, influenced by the caregivers’ own gender role expectations, thus modulating infants’ opportunities to be exposed to talking faces. For example, some studies have found that female infants receive more vocal initiations—i.e., a greater total number of seconds of talking, laughing, or making noises—from their fathers than their male counterparts (Brundin et al., 1988), higher frequency of mothers’ vocalization than their male peers (Lewis, 1972), and more language input overall [though evidence is mixed; see Bergelson et al. (2019)]. Thus, one possibility is that female infants have increased exposure to audiovisual speech input from their caregivers compared to their male peers, providing more chances to learn to scan social events efficiently.

Alternatively, caregivers may talk more to infants with more advanced language skills—which tend to be primarily females (Eriksson et al., 2012). For example, in a longitudinal study that collected home recordings between 6 and 17 months, Dailey and Bergelson (2022) replicated the finding of larger vocabularies in females than males but found no sex differences in parents’ language input. Instead, caregivers talked more to infants who had begun to talk, regardless of sex. We speculate that more talkative infants—who tend to be primarily females—may receive more face-to-face audiovisual speech input during interactions, thereby enhancing their opportunities to learn linguistic information from this event. In support of this possibility, Lavelli and Fogel (2002) found that 3-month-old female infants spent more time than males in face-to-face communication with their mothers.

#### 3.2.2 Caregivers’ adaptations to infants’ neurocognitive functioning

Caregivers may also adapt to their infants’ neurocognitive functioning. In neurotypical development, infants with better attentional skills to social and non-social events engage in more social contingency during interactions with caregivers and, reciprocally, social contingency supports infant**s’** attention and language outcomes (Masek et al., 2024). If, in the first months of life, female infants, at a group level, develop neural networks that are more efficiently responding to complex social events (e.g., DMN, Salience Network) than those of males, this may lead them to select and seek more social information (including audiovisual speech) than male infants. This differential responsiveness in infants could lead caregivers to be more verbally responsive to females, potentially enhancing their ability to visually scan audiovisual speech. Some indirect evidence points to this possibility. For example, Sung et al. (2013) found that while mothers of female infants gradually increased their contingent responsiveness to their infants’ vocalizations across the first year of life, mothers of male infants did not.

Although the literature on this topic in EL-infants is still scant, some recent studies with younger siblings of autistic children (aged 2–3 years) have found sex differences in conversational skills in caregiver-child interactions (Britsch and Iverson, 2024). Across autism likelihood groups, males produced more adjacent utterances than females (within 5 s following the onset of a caregiver utterance), while females produced more contingent utterances that added new information to the conversation (i.e., related to the ongoing topic). Further, caregivers of females produced longer utterances than caregivers of males. Although further studies are needed with infant siblings, these findings suggest that at least some environmental mechanisms placed at the level of dyadic interactions may be modulated by infants’ biological sex across neurotypical and at-elevated-likelihood-for-autism infant populations.

Because infants’ learning to selectively attend to the articulating mouth depends on their visual attention skills, their access to audiovisual speech input, and the social contingency in caregiver-child interactions, a promising direction for future research would be to examine with ‘live’ eye-tracking whether female infants receive more audiovisual speech input than their male peers (or if the more advanced infant talkers do, regardless of sex). Additionally, it would be valuable to investigate how the amount of exposure to this event and social contingency with caregivers could modulate mouth-looking trajectories and later language outcomes by sex.

#### 3.2.3 Cultural influences

Language-specific features and more global social practices of interaction that extend beyond the level of infant-caregiver dyads may also affect the trajectories of mouth-looking. However, to our knowledge, few studies have compared linguistically and culturally diverse samples, as most research has followed an Anglocentric approach (see Bastianello et al., 2022, for a systematic review). For example, Sekiyama et al. (2021) observed that Japanese language-learning infants and toddlers preferentially attended to the eyes instead of the mouth when exploring talking faces, contrasting with previous findings in English language-learning populations, who preferentially attended to the talker’s mouth. The authors interpreted this finding as reflecting a potential effect of the limited involvement of visual articulatory information and the larger contribution of tonal information in Japanese.

Potential cultural differences in selective attention to the articulating mouth could also interact with sex differences in infancy and toddlerhood. For example, in a cross-cultural study of toddlers by Lozano et al. (under review)^2^, sex differences were not replicated across cultures. While Norwegian male toddlers attended more to the mouth than their female peers, this was not the case for Polish toddlers. Perhaps how much caregivers’ practices are influenced by sex/gender expectations in a given culture may partially account for these differences. Alternatively, the properties of a given language may shape a different timeline of sex differences in the trajectories of mouth-looking across cultures. This highlights the need to interpret potential sex differences in mouth-looking within the context of the infants’ own cultural developmental trajectory, which is particularly important in infant siblings’ populations.

If sex differences in mouth-looking are accounted not only by biological factors but also sex/gender-driven cultural and caregivers’ practices, and infants’ previous linguistic experience, we may find that sex differences in mouth-looking emerge at different timepoints across cultures. Further understanding potential differences and similarities of this trajectory’s time scale across cultures may help us avoid interpreting a deviation from the typical trajectory of mouth-looking as a difference in infant siblings when it may actually reflect the impact of cultural factors.

## 4 Future challenges: Methodological and theoretical unsolved issues

### 4.1 Methodological

Despite the growing evidence on mouth-looking as a potential early marker of language acquisition, challenges remain across neurotypical, autistic, and at-elevated-likelihood for autism populations. These challenges are both methodological and theoretical, and addressing them is crucial because how we measure and interpret the marker itself could impact what sex differences in mouth-looking might mean in the general population and in the context of autism. Although we will discuss these issues separately for the sake of clarity, we believe they are closely intertwined.

Methodologically, a significant first issue is that current evidence is correlational, leaving the relation between increased mouth-looking and better language outcomes unclear. While causal evidence would allow us to robustly consider mouth-looking as an early marker—*directly* influencing language—, correlational data leave open the possibility that other unmeasured mediating factors are at play. For example, more looking at the mouth in itself may not directly improve language skills but rather the extent to which infants *benefit* from looking at the mouth. This is because simply looking at the mouth does not guarantee that infants will *learn* linguistic information from the mouth, such as phoneme categories. We suggest that it may be possible to measure the benefit of infants’ looking at the mouth and eyes of a talking face for language development. For example, we could manipulate infants’ looking time to these regions and measure its impact in skills that mediate the relation between infants’ mouth-looking and later language development, such as audiovisual speech integration (Kushnerenko et al., 2013a, 2013b). This could be tested by comparing infants’ performance in a McGurk paradigm (in audiovisual syllables) across three conditions: cueing the mouth, cueing the eyes, and free-viewing [see Feng et al., 2023, where this design was used in autistic and neurotypical children]. If increased mouth-looking enhances audiovisual speech integration, then it is expected that cueing the mouth would improve infants’ performance relative to the other two conditions. To investigate the benefits of increased mouth-looking in audiovisual *fluent* speech, we could measure infants’ cortical tracking of speech while presenting talking faces with the mouth occluded (i.e., mouth covered) versus visible (i.e., a free-viewing talking face). Two control conditions could be included, as in Haider et al. (2024): auditory-only speech, to rule out effects due to acoustic degradation, and a visual-only speech (a silent talking face) to control for effects of visual features of the talking face alone. If mouth-looking improves infants’ processing of audiovisual fluent speech, then we expect greater cortical tracking accuracy in the visible mouth condition compared to all other conditions.

Related to the first issue, it has been often assumed only one ontogenetic directionality across development in correlational studies; looking more at the mouth in the second half of the first year provides important cues that lead to better later language development. However, the opposite may be the case; during this period, infants who have higher language skills may look more at the mouth, possibly because they benefit more from doing so. This increased looking at the mouth may help them to develop better language later on in toddlerhood, regardless of how much they look at the mouth at that time. Another possibility is that the same underlying factors (e.g., infant-caregiver’s joint engagement, infants’ vocal complexity; Santapuram et al., 2022) influence both mouth-looking and language development, but that mouth-looking does not in itself lead to better language outcomes. To address this, experimental manipulation of mouth-looking and the examination of other related mechanisms are necessary. Furthermore, longitudinal research using cross-lagged models could also be useful in exploring the relations (and their directionality) between mouth-looking, language development, and other variables over time.

A third unsolved issue is whether looking at the eyes and mouth are *methodologically* independent. Previous studies have used various metrics to measure preference for the eyes and mouth, often leading to inconsistent results. Some use absolute measures like the proportion of total looking time (PTLT) to the eyes or mouth relative to the whole face (so-called PTLT Eyes and PTLT Mouth). Others use relative measures, such as the eyes-mouth index (EMI index – calculated as the mean amount of gaze to the eyes relative to both the eyes and mouth; e.g., Viktorsson et al., 2023), or a PTLT difference score (PTLT Eyes minus PTLT Mouth; e.g., Morin-Lessard et al., 2019). Absolute measures factorize eyes and mouth as ‘areas of interest’, calculating PTLT independently for each and treating PTLT as a dependent variable (e.g., Lewkowicz and Hansen-Tift, 2012). In contrast, relative measures consider preference for the eyes and mouth as a single dependent variable. These different metrics might not only reflect different calculations but also represent different constructs, and imply varying degrees of interdependence between eyes and mouth-looking. Linking absolute measures of mouth-looking with language outcomes could offer a more direct approach to identifying whether mouth-looking is an early language marker, as relative measures may obscure potential trade-offs between infants’ selective attention to eyes and mouth.

Fourth, it is necessary to understand how generalizable mouth-looking could be as a potential female-specific marker of later language development across contexts (experimental vs. real life). One potential solution for future studies would be to use ‘live’ eye-tracker (vs. screen-based), which allows tracking of infants’ eye-gaze patterns to talking faces during interactions with real people instead of recorded clips of talking faces. Further, specifically, dual ‘live’ eye-tracker would enable tracking caregivers’ eye-gaze to infants and mutual gaze between both. This method not only facilitates the investigation of potential sex differences at the infant level (e.g., sex-specific patterns of selective attention to the mouth) but also at external levels (e.g., language input) and internal-external levels (e.g., infant-caregiver interactions). Furthermore, if sex differences in selective attention to the mouth of talking faces are influenced by other domains (e.g., sex differences in infants’ motor development), these new tools can provide insights into such relations.

Fifth, tracking potential sex differences in mouth-looking across developmental time will be important, as these differences may not be stable but could emerge only during certain periods. Longitudinal studies tracking these differences from the first months of life until toddlerhood will be necessary to obtain a comprehensive developmental picture and assess the impact of both initial biological influences and infants’ experiences on them.

A final methodological challenge is to test the stability of sex differences in mouth-looking across countries, which will inform us about the robustness of increased mouth-looking as a female-specific early marker of language. It will reveal potential similarities and differences in infants’ developmental trajectories of mouth-looking across various sites, highlighting the relative contributions of mechanisms at a biological and environmental level. Collaborative cross-lab research between infant labs is crucial for investigating that and achieving high-powered sample sizes that allow balanced comparisons between sexes. In particular, future collaborations consisting of secondary analyses of existing eye-tracking data with already established consortia could help to overcome this challenge. Examples include potential collaborations with the Eurosibs consortium (Jones et al., 2019), which involves researchers from five European countries (the UK, Sweden, Belgium, Poland, and the Netherlands), the research network and funding consortium British Autism Study of Infant Siblings (BASIS; https://www.basisnetwork.org/), or the Baby Siblings Research Consortium (BSRC)—a network of over 40 scientists worldwide. All of these consortia have already conducted large cohort longitudinal studies on sex differences in infant siblings of autistic children (e.g., Bedford et al., 2016; Messinger et al., 2015; Siqueiros Sanchez et al., 2021), which could be extended to the study of sex differences in mouth-looking in this population and neurotypical peers. Importantly, incorporating infant siblings from new entities outside these existing consortia will also be crucial for increasing representativeness across sites.

### 4.2 Theoretical

At a theoretical level, it remains unclear whether infants’ preferences for the eyes and mouth when observing talking faces are *mechanistically* independent. It has been commonly assumed that eyes-looking is driven by socio-emotional or referential cues, while mouth-looking is driven by visual speech cues or infants’ growing understanding of the linguistic content (Bastianello et al., 2022; Çetinçelik et al., 2021). Some longitudinal studies suggest some level of independence, as eyes and mouth-looking independently change across development; while eyes-looking remained stable across the first year, mouth-looking increased (e.g., Lozano et al., 2022). However, other longitudinal evidence points to a functional overlap. For instance, increased looking at the eyes in 5-month-olds prospectively relates to higher language comprehension at 14 months (Viktorsson et al., 2023) and to both language comprehension and production at 24 months (Lozano et al., 2022). Furthermore, both increased mouth-looking in the second half of the first year and the magnitude of gains in mouth-looking during this period are associated with better expressive and receptive language outcomes in toddlerhood (Lozano et al., 2022; Tsang et al., 2018; Viktorsson et al., 2023). This suggests that both eyes and mouth-looking might be mechanistically related as early markers of language development; eyes-looking might be more important early on, while mouth-looking becomes increasingly important during the last half of the first year. Furthermore, this evidence highlights the need to distinguish productive and receptive language outcomes.

**Figure 2:**
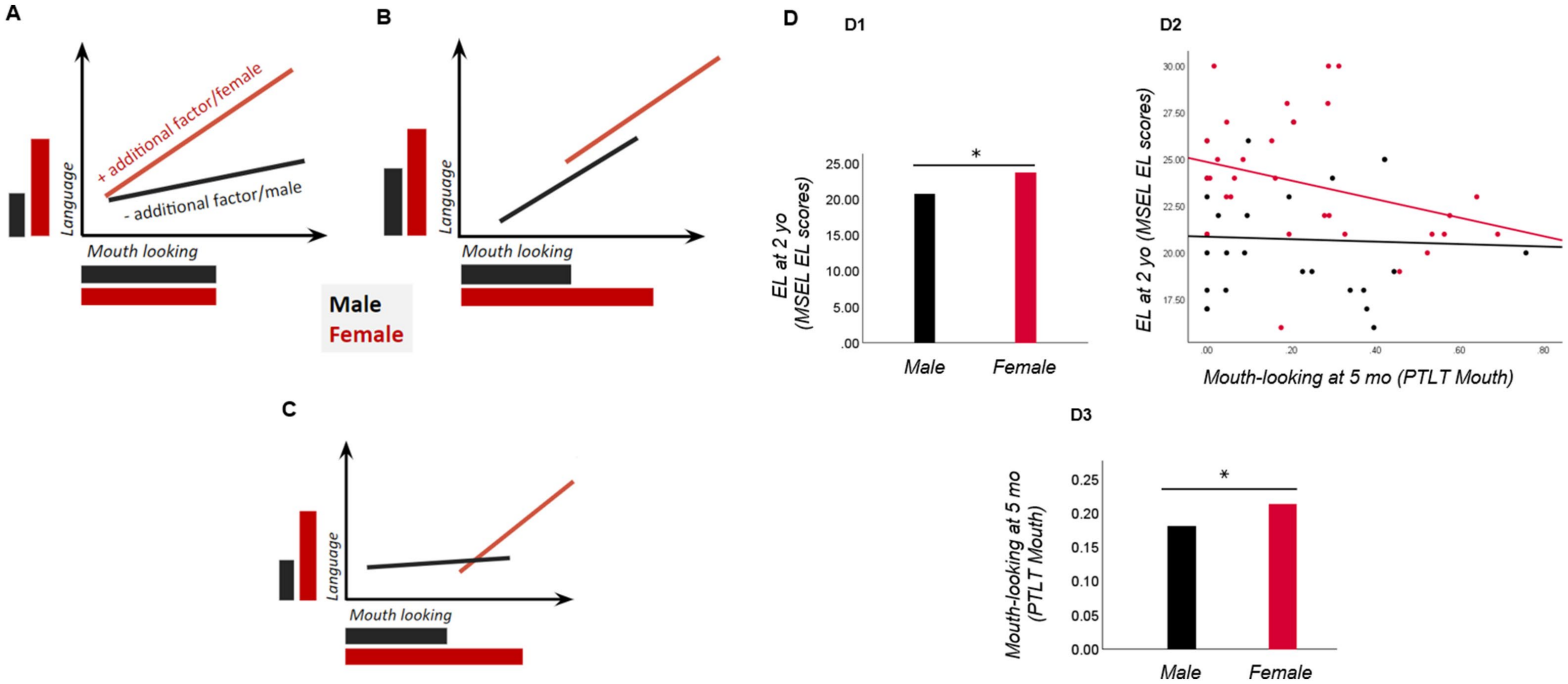
Different sex differential models link mouth-looking as an early marker to later language outcomes. **(A)** The early marker (mouth-looking) is similarly expressed in both sexes but relates to better language outcomes only in females. This may reflect sex-specific additional factors (unidentified) that make mouth-looking more effective in females; **(B)** Sex differences display in mouth-looking, but the marker relates to better language similarly in both sexes; here, higher mouth-looking levels protects language development in females, but a negative relation may also occur; **(C)** A combined model is also possible: a non-linear association between mouth-looking and language (where a certain amount of mouth-looking is needed for an association with language to be seen), combined with sex differences in mouth-looking. **(D)** Actual data from neurotypical infants (plotted with permission from Lozano et al., 2022) show sex differences in the early marker (more mouth-looking in females than males; **D3**), expressive language outcomes (higher in females; **D1**), and their associations (negative, and female-specific; **D2**). Mo: months. Yo, years; EL, expressive language; MSEL, Mullen scales of early learning; PTLT, proportion of total looking time.

A second key theoretical issue is whether increased selective attention to the mouth serves as a sex-specific protective marker for language development. In Figure 2, we set out several sex differential models (following a similar logic to Bedford et al., 2016) that would apply to both EL and LL-infants, and plot actual data in neurotypical infants. First, if only female infants, regardless of autism likelihood, show increased mouth-looking, and mouth-looking predicts better later language development only in females, then we would interpret it as an early marker acting as a female-specific protective marker for language acquisition (in both female LL and EL-infants). Note that this relation could be either positive or negative, but female-specific (see more below). Alternatively, if there are no sex-differences in mouth-looking itself, but only in its relation to language outcomes, with the association between looking at the mouth and language skills (negative or positive) occurring only in females (LL and EL infants), this would also suggest that mouth-looking acts as a female-specific protective marker. This could imply that while both sexes look at the mouth equally, another (unidentified) factor might make females use this information more effectively, and mouth-looking therefore is associated with later language only in females. Second, if increased mouth-looking is displayed by both sexes, regardless of autism likelihood status, and mouth-looking predicts better language outcomes regardless of sex, we would see it as an early precursor of better language development. A final possibility is a combined model: a non-linear association between mouth-looking and language, with sex differences in mouth-looking.

A third theoretical issue is interpreting the direction of the potentially female-specific relations between mouth-looking and language outcomes. Lozano et al. (2022) found that females looked more at the mouth than males in the first year but showed a negative relation between mouth-looking and language outcomes. We speculated above that this finding could potentially suggest an early native-language expertise in females, as infants with better language might need to rely less on the mouth to seek visual articulatory speech cues (Lewkowicz and Hansen-Tift, 2012), and perhaps an earlier peak in mouth-looking in females, before 5.5 months. Longitudinal studies could clarify potential sex differences in the timing of mouth-looking and perceptual narrowing, and whether the female-specific associations may shift from positive early on (before 5.5. months) on to negative later in development. Relatedly, we need to develop clearer theoretical criteria to distinguish whether the absence of a protective marker in males that is present in females (i.e., the absence of longitudinal relations between mouth-looking and language outcomes in males) is equivalent to the presence of a risk marker in males. Alternatively, it may be more accurate to say that mouth-looking confers protection in females but not in males, but we need specific thresholds for this. At a broader level, it remains an unresolved challenge whether it is even possible to distinguish clearly between risk and protective factors, as this requires an assessment of causality (Johnson et al., 2021); this is why we have preferred to refer to *markers* in this paper.

Finally, tracking potential sex differences in mouth-looking across developmental time will be important, as these differences may not be stable but could emerge only during certain periods. Longitudinal studies tracking these differences from the first months of life until toddlerhood will be necessary to obtain a comprehensive developmental picture and assess the impact of both initial biological influences and infants’ experiences on them. In addition, it would be particularly valuable to investigate potential sex differences in mouth-looking and their links to language development not only in the second half of the first year of life but throughout the first two years of life, to capture potential links with multiple language skills, including pre-linguistic skills such as canonical babbling and the production of word approximations.

## 5 Conclusion

Females are more likely than males to exhibit higher language skills across both autistic (Harrop et al., 2021; Lai et al., 2015) and neurotypical populations (Eriksson et al., 2012) during early and mid-childhood (0–11 years). Neurotypical males start to catch up with female peers after age 6 years (Bornstein et al., 2004), but this phenomenon remains inconclusive in autistic males (e.g., Nishimura et al., 2023). What developmental mechanisms underlie this difference between sexes in early language acquisition? One of the possible answers lies in how females visually attend to the articulating mouth of talking faces from early on in life, which may potentially enhance their ability to extract linguistic information efficiently, thereby providing them with an advantage in later language acquisition compared to males.

Drawing from existing evidence on sex differences in language acquisition and social attention in neurotypical infants and young autistic individuals, in this article we have outlined the hypothesis that selective attention to the articulating mouth during infancy may be a female-specific early marker that may serve as a sex-specific protective marker for language acquisition in populations at elevated likelihood for autism. The implications of our hypothesis are that: (1) sex differences in this attentional mechanism of language acquisition may be shared by young autistic children (Harrop et al., 2018), EL-infants (Chawarska et al., 2016; Kleberg et al., 2019), and neurotypical infants (Alexander and Wilcox, 2012), following a continuous distribution across autistic individuals and the general population; (2) females, at a group level, are likely to show a more efficient trajectory of selective attention to the mouth than males across these populations; and (3) this protective effect on language development is also expected to be shared among young autistic females, female EL-infants, and neurotypical infant females, at a group level. Ultimately, we propose that sex may override or enhance genetic risk markers linked to autism and its associated language differences by modulating this attentional mechanism.

We have also reviewed behavioral, neural, genetic, and hormonal evidence pointing to potential mechanisms placed at the biological and environmental levels that may account for sex-differentiated neurodevelopmental pathways of mouth-looking and language development. Evidence suggests that sex differences manifest at a group level in several of these mechanisms already in utero and likely extend through the lifespan. We believe that there are likely to be sex-specific pathways of language acquisition along a continuum, with potential individual differences within sexes also being plausible. Several of these mechanisms where sex differences are present (e.g., functional connectivity in social brain networks, testosterone levels, and language input from caregivers) play critical roles in social cognition development. This speaks for the possibility that differences in some socio-communicative skills (e.g., language acquisition) typically observed in autism may disproportionately affect males than females from early on, potentially cascading in male-specific liabilities in these domains across neurotypical and autistic populations. Weighted cumulative risk and protective markers in other mechanisms of social development may contribute to typical or different trajectories of language acquisition in infant males. Notably, sex differences were also evident across autism likelihood status, suggesting continuity in the mechanisms underlying language development in infants with both neurotypical and potentially different trajectories.

## Data Availability

Publicly available datasets were analyzed in this study with the authors’ permission. This data can be found at: https://osf.io/t93x8/.

## Footnotes

^1^Here, we understand gender as an umbrella term that refers to socially constructed characteristics of women and men, including behaviors, roles, activities, and attributes that a given society considers appropriate for each of them (WHO, 2024).

^2^ Lozano, I., Duszyk-Bogorodzka, A., Sophie Ribu, I., Laudańska, Z., Szmytke, M., Dynak, A., et al. (under review). The role of attention to the mouth of talking faces for vocabulary skills during toddlerhood: does language familiarity still matter? Manuscript submitted for publication.
